# Benchmarking ANI potentials as a rescoring function and screening FDA drugs for SARS-CoV-2 M^pro^

**DOI:** 10.1007/s10822-024-00554-4

**Published:** 2024-03-27

**Authors:** Irem N. Zengin, M. Serdar Koca, Omer Tayfuroglu, Muslum Yildiz, Abdulkadir Kocak

**Affiliations:** 1 Department of Chemistry, Gebze Technical University, 41400 Gebze, Kocaeli Turkey; 2 Department of Molecular Biology and Genetics, Gebze Technical University, 41400 Gebze, Kocaeli Turkey; 3 Present Address: Pfizer - Universidad de Granada - Junta de Andalucía Centre for Genomics and Oncological Research (GENYO), 18016 Granada, Spain

**Keywords:** Molecular docking, Scoring function, Interaction energy, Binding mode, Rescoring

## Abstract

Here, we introduce the use of ANI-ML potentials as a rescoring function in the host–guest interaction in molecular docking. Our results show that the “docking power” of ANI potentials can compete with the current scoring functions at the same level of computational cost. Benchmarking studies on CASF-2016 dataset showed that ANI is ranked in the top 5 scoring functions among the other 34 tested. In particular, the ANI predicted interaction energies when used in conjunction with GOLD-PLP scoring function can boost the top ranked solution to be the closest to the x-ray structure. Rapid and accurate calculation of interaction energies between ligand and protein also enables screening of millions of drug candidates/docking poses. Using a unique protocol in which docking by GOLD-PLP, rescoring by ANI-ML potentials and extensive MD simulations along with end state free energy methods are combined, we have screened FDA approved drugs against the SARS-CoV-2 main protease (M^pro^). The top six drug molecules suggested by the consensus of these free energy methods have already been in clinical trials or proposed as potential drug molecules in previous theoretical and experimental studies, approving the validity and the power of accuracy in our screening method.

**Supplementary Information:**

The online version contains supplementary material available at 10.1007/s10822-024-00554-4.

## Introduction

Designing a therapeutic molecule to treat a disease can be quite expensive and time consuming. Preclinical processes such as identification of drug targets, High Throughput Screening (HTS) experiments on the target, generating a library of hit compounds, optimizing potency of bioactive compounds and preceding clinical trials require tremendous amount of resources. Typically, a drug can cost more than 2 billion US dollars and 10–15 years to be approved and enter the market [1–3]. Since repurposing an FDA approved drug is a much faster and less expensive strategy it has become one of the most popular approaches in recent drug development endeavors. Even testing all FDA approved drugs experimentally may not be affordable in terms of economic burden and time. To help ease this problem, computational methods have been utilized in rational drug design.

In searching for drug candidates as potential inhibitors, binding mode and binding affinity are two critical questions that ought to be addressed. Docking methods are capable of screening ultra large libraries of compounds by aforementioned approaches (billions of molecules) with much reduced cost and time. Docking methods rely on a search algorithm and scoring function that are physics based, empirical, knowledge-based and Machine Learning based which compromise on accuracy to increase the speed [1, 4, 5]. Despite the great success of the docking methods, there is still a need for improving scoring functions so as to reproduce experimental results.

Machine Learning (ML) methods have just started to gain attention to advance the docking methodology recently similar to other computational techniques. The main objective of the ML techniques is to find an accurate and a fast solution to an existing problem by learning from previous experimental data. This is achieved by supervised, unsupervised and reinforcement methods [6]. Supervised methods are based on training an algorithm on a set of inputs to find an output while unsupervised learning algorithms find a pattern in data set predict results from this arrangement. On the other hand, reinforcement learning, the input progress in an environment and uses the data learned from the experience [1]. Recent studies have shown the outperformance of ML based scoring functions over classical ones, which directly use a known mathematical function [7–9]. ML based scoring functions are usually used by means of rescoring [10] due to the dependence on the training dataset [11, 12].

ANI-1, ANI-1x and ANI-1cxx have been trained to calculate DFT and CCSD(T) energies of small organic compounds containing C, H, O and N atoms in non-equilibrium conformations [13–15]. The extended version, ANI-2x, has been shown to predict DFT energies of equilibrium or non-equilibrium conformations of molecules containing C, H, O, N, S, F and Cl atoms. It has been shown to reproduce the energies at the accuracy of the ωb97x/6-31G* level with millions of times faster than the actual QM calculations [16].

The severe acute respiratory syndrome coronavirus 2 (SARS-CoV-2) is the cause of the coronavirus disease pandemic in 2019 [17]. Since the first case of COVID-19 in 2019, more than 6.8 million deaths have been reported worldwide (World Health Organization. Weekly Epidemiological Update on COVID-19. [18]). The coronavirus disease 2019 (COVID-19) pandemic, caused by severe acute respiratory syndrome coronavirus 2 (SARS-CoV-2), has destroyed health systems, societies, and economies. After the first identification of the SARS-CoV-2 strain coming from Wuhan, several variants of concern (VOCs) have been identified. Clinical reports and epidemiological features of the infection indicate a relatively mild disease flow and increased human-to-human virus spread [19–21]. Improved COVID-19 treatment and prevention techniques are urgently required given the increasing SARS-CoV-2 infection rate and lack of efficient treatment options. Researchers around the world are working to develop treatments and vaccines to struggle the disease, and several drugs have been approved for emergency use by regulatory agencies. For now, only two SARS-CoV-2 oral drugs (Paxlovid and Molnupiravir) have been clinically approved [22, 23]. Nevertheless, the antiviral potency of these compounds is not adequate to deal with the pandemic. Paxlovid (Nirmatrelvir with Ritonavir) has been reported to permit the SARS-CoV-2 replication upon completion of 5 day long oral take [24]. In addition to limited number of vaccines and drugs, the emergence of drug resistant variants of SARS-CoV-2 brings additional concerns [24, 25] about the use of those drugs [26]. Therefore, developing anti-viral oral therapeutics for COVID-19 is still highly demanded.

Similar to other viral infections, once entering the host cell cytoplasm, the SARS-CoV-2 viral genome is translated into approximately 30 proteins. At first, 16 of these proteins are translated as two polyproteins, and in order to continue infection, these polyproteins must be split apart into the two proteins that virally encoded proteases; the major viral protease known as 3CL^pro^ (or M^pro^), and the protease papain-like protease (PL^pro^), facilitate the cleavage of these polyproteins into structural and non-structural proteins (NSPs) [27], which play a crucial function in the transcription/replication during the infection. Since Mpro is a critical enzyme in the life cycle of the virus [28], it has been a validated high-profile antiviral drug target, and its inhibitors have been shown to have strong antiviral activity in cell cultures and animal models [22, 29–37]. Other coronavirus enzymes, like the helicase and the RNA-dependent RNA polymerase, has potential to be target for the development of antiviral drugs, such efforts are currently limited because these enzymes don't have crystal structures [37, 38]

Main protease (M^pro^) is a three-domain (domains I–III) cysteine protease consisting of 306 amino acids. Anti-parallel β-barrel structures takes place domain I and domain II (residues 8–101/residues 102–184, respectively), and domain III (residues 201–303) is connected to domain II through a long loop region (residues 185–200) that contains 5 α-helices arranged in one substantially antiparallel globular cluster [28]. The glutamine residue in the P1 position of the substrate is cleaved by Mpro using the protease Cys145-His41 dyad, where the cysteine thiol serves as the nucleophile in the proteolytic reaction.

Designing a therapeutic molecule from the ground up to treat an illness can become extremely costly and a time taking process. In particular, for pandemics with such high transmission rates like SARS-CoV-2, requires prompt actions for controlling the disease. Instead, drug repurposing strategy might offer inexpensive and faster solutions to the problem. Repurposed drugs that have existing clinical data on the effective dose, treatment duration, side effects, and toxicity could be rapidly translated into the treatment of patients.

High throughput virtual screening by means of drug repurposing have been used to identify safe-in-human drugs with potential anti–SARS-CoV-2 properties. Most of these studies are based on molecular docking. Although it is the gold standard method to find the binding mode, docking is a very coarse method and almost never predicts the correct experimental binding affinity trend among the inhibitors. One strategy to overcome this limitation is to use consensus scoring [39]. More sophisticated methods to calculate the potential binding free energy of inhibitor candidate to the protein ranges from post molecular dynamics simulations such as Molecular Mechanics Poisson-Boltzmann Surface Area (MM-PBSA) to perturbation methods such as Bennett acceptance ratio (BAR), the latter being much more accurate yet quite costly. Although there have been numerous attempts that combine docking and MD based free energy methods in virtual screening, the number of candidates is mostly reduced to tens of hit compounds prior to MD simulations in these studies due to computational cost. Therefore, the top pose from docking is usually used in MD simulations and free energy calculations. Although different docking algorithms are successful in prediction of correct binding mode in the top three poses [40], MD simulations towards screening are performed only on the first pose. This limits the success of the binding free energy calculations (BFE) when the ligand is totally mis-oriented (such as flipped) among the top poses in docking since one cannot expect the MD simulations’ correcting these drastic changes no matter how long the simulations performed. Therefore, running separate MD simulations for all the top poses may become a necessity to correctly predict BFEs.

One-trajectory approach end-state BFE calculations are quite attractive since they require only one MD simulation for each protein−ligand (PL) complex system with less computational cost. Although relatively better than docking scores, the accuracy in end-state BFE methods is still low due to over-simplifications such as implicit solvent definition in the case of MM-P(G)BSA and molecular mechanics (MM) definition of the Hamiltonian of the system. On the other hand, several new implementations have been introduced to end-state methods to improve accuracy. We have recently implemented the use of ML based potentials as a post MD simulation to improve the accuracy of BFE calculations [41, 42]. Herein, we introduce a unique virtual screening protocol to overcome aforementioned drawbacks.

Here, we first investigate and unravel the capability of ANI potentials as a rescoring function in molecular docking. Our results show that the docking power of ANI potentials can compete with the current scoring functions at the same level of computational cost. We then screened a library of 2500 clinically used drugs, either approved for human use or with extensive safety data in humans (phase 2 or 3 clinical trials), for their ability to bind SARS-CoV-2 Mpro using consensus docking scores of ANI/GOLD and free energies by MMPBSA/ANI_LIE.

## Computational methods

### CASF-2016 dataset

In order to test ANI’s performance as a rescoring function, we have used CASF-2016 dataset, which has been created for benchmarking purposes in docking algorithms. The dataset is composed of 285 protein–ligand complexes and each complex has a native binding pose as well as maximum 100 decoy ligand binding poses, selected from a normal distribution of 1000 poses generated by three different docking software with RMSD ≤ 10 Å from the crystal structures [40, 43, 44]. In the study, 34 different docking algorithms/software were tested in terms of “ranking power”, “scoring power”, “docking power” and “screening power”. Docking power is defined as whether a scoring function can properly differentiate the native binding pose from all the decoys within the top 1,2, or 3 scores. In addition, when the different decoys create a potential energy surface in the binding region, the native pose should correspond to the minimum energy structure. This has been defined as “binding funnel analysis” and evaluated based on Spearman’s rank correlation between the 10 bins of different RMSD windows and average scores for these bins [40]. We used the docking poses provided in this CASF-2016 dataset directly in assessment of the performance of ANI-2x. We have calculated the binding affinities by ligand interacting interacting with only residues in docking region (grid box/sphere) by saturating the discontinuing atoms with hydrogens using Pdbfixer [45].Since the ANI-2 × has been trained for molecules containing only H, C, O, N, S, F, and Cl atoms, the total the dataset reduced to 254 from the original set of 285 proteins. In addition, we also tested the ANI’s performance on SARS-CoV-2 main protease inhibitors by applying our own docking protocol using GOLD software.

### GOLD docking protocol

The inhibitors of SARS-CoV-2 main protease that are reported in Protein Data Bank (PDB) were retrieved (complex PDB IDs: 7N44, 7L10, 7L11, 7L12, 7L14, 7M8M, 7M8P, 7M8O, 7M8N, 7M8X, 7M8Y, 7M90, 7M8Z, 7M91) and docked to the protein structure with PDB ID: 7L14. Docking was performed using the GOLD Suite v.5.3 software by Cambridge Structure Database (CSD) [46]. For GOLD, the ChemPLP scoring function was used since it is known to give better results in prediction of binding mode than other scoring functions implemented in the software [26]. The genetic algorithm with a minimum of 1,000,000 and maximum 1,250,000 iterations was used. A grid sphere of 10 Å radius was defined. The center of the sphere was defined by the reference ligand in the structure of 7L14. All rotatable bonds in protein were frozen while the ligand was defined as flexible. The bond orders, hydrogens, atom types, and partial charges were produced by the Hermes software’s edit utilities. GOLD software was constructed to produce 25 poses for each ligand without early termination.

### ANI-2× as rescoring

ANI scoring relies on the interaction energy between the receptor and the ligand similar to force field based scoring functions. The interaction energy is calculated by ANI-ML potentials rather than non-covalent coulombic and van der Waals interactions of conventional force field based scoring. The details of the calculation can be found on https://github.com/otayfuroglu/deepQM and the related ANI_LIE work by Akkus et al.[41], as will be discussed in the next section. The main difference is to use docking pose instead of calculating frames from an MD simulation trajectory. Apart from the input pdb file with protein and ligand complex, the user is required to give an index file containing the index groups to be calculated. The index file is the one produced by Gromacs (i.e., listing comma separated residue numbers followed by the group name in the square bracket). The ligand and the residues in the docking region (or entire protein) are defined as separate groups in the index file. Thus, ANI scores can be readily calculated as the difference in energy between the index groups corresponding to complex and free components (protein and ligand) so that $\Delta {E}_{ANI}={E}_{complex}-({E}_{protein}+{E}_{ligand})$. The interaction energy $\Delta {E}_{ANI}$ is then translated to free energy using a scaling factor, as discussed in the next section. Using this approach, each pose reported on CASF-2016 dataset or generated by our docking with GOLD was recalculated by ANI scoring function.

### MD simulations

A similar protocol to our previous works has been used in MD simulations [41, 42, 47–53]. Briefly, ligands were first optimized at B3LYP/6-31++G(d,p), and Merz–Kollman (MK) electrostatic potential (ESP) charges were computed at HF/6-31G* level using Gaussian 16 software. GAFF2 parameters and restricted electrostatic potential charges (RESP) were generated using the antechamber utility in AmberTools 22. The amber99sb-ildn force field was used for the protein's topology. Solvation of protein–ligand complexes employed the TIP3P water model in a dodecahedron box with 10 Å dimensions for each axis. System neutralization occurred at a salt concentration of 0.15 M Na+ and Cl− ions. Energy minimization, utilizing the steepest descent algorithm with a Verlet cutoff scheme, was performed to a maximum force of 100 kJ mol^−1^ nm^−1^. Electrostatic interactions were computed using the particle-mesh Ewald (PME) method, while bonds involving hydrogen atoms were constrained with a harmonic potential. Prior to the final simulations, a 5 ns NVT-MD equilibration at 310 K was conducted using a Langevin thermostat followed by 200 ps and 1 ns NPT-MD equilibrations at 1 atm using Berendsen and Parrinello-Rahman barostats, respectively.

### Free energy calculations

The single-trajectory approach MMGBSA calculations were calculated using the gmx_MMPBSA [54]. The default parameters with the internal dielectric constant, ε_int_ = 2 for polar solvation terms. The SASA-only model was applied with parameters of γ = 0.0072 kcal/mol·Å^2^ and b = 0 for nonpolar terms.

In addition to MM-PBSA calculations, we also used ANI_LIE, a recent end-state binding free energy method introduced by Akkus et al. [41]. The details of the method has been discussed elsewhere [41]. Briefly, the method uses the linear interaction energy (LIE) approach utilizing the potential energies predicted by ANI-ML potentials as a replacement to molecular mechanics (MM) energy terms in LIE formalism. The free energy is calculated by:

$$\Delta {G}_{bind}=\beta {\langle \Delta {E}_{ANI}^{L-P}\rangle }_{PLS}+\gamma$$

where ${\langle \Delta {E}_{ANI}^{L-P}\rangle }_{PLS}$ is the average interaction energy between the protein and ligand produced by extracting the MD frames for energy groups of protein (P), ligand (L) and protein–ligand (PL).

For ANI_LIE calculations were performed using the simplest form neglecting the D3 term and solvation effects (i.e., Eq. 4 in Ref. [41]) with default $\beta$ and $\gamma$ empirical parameters of 0.127 and −5.11, respectively. As reported by Akkus et al. [41], these parameters were optimized SARS-CoV-2 M^pro^ and have been successfully used in other studies [42, 47, 48, 55, 56].

## Results and discussion

### Benchmarking with other docking software on CASF-2016 dataset

**Fig. 1:**
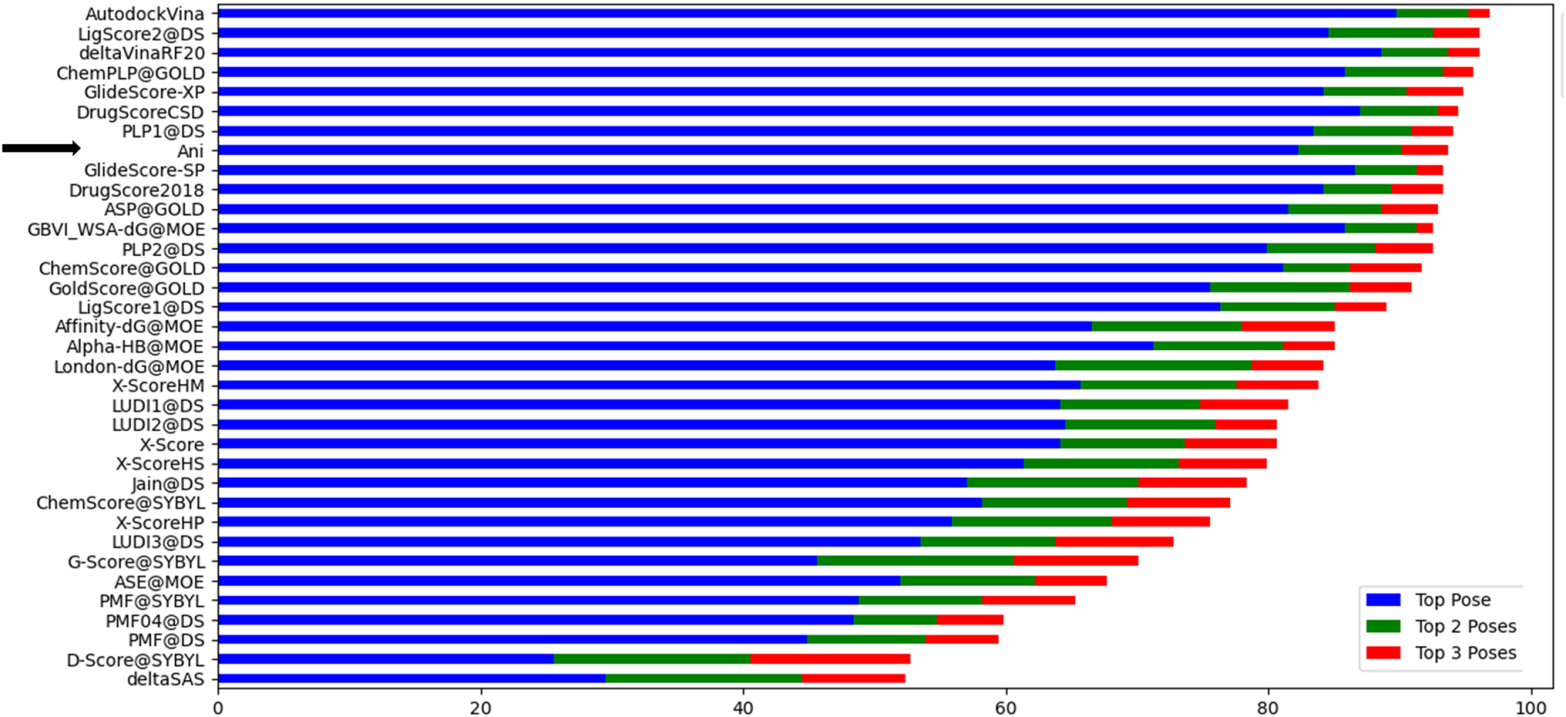
The success of different scoring functions on predicting the first poses as the closest structure to the crystal with RMSD less than 2.0 Å. ANI is ranked 8th among 34 functions

In the first part, we tested the scoring produced by ANI predicted interaction energies on a standard dataset containing diverse protein–ligand complexes. Using ANI, we have rescored all the poses belonging to 254 protein–ligand complexes and compared to the results of the original publication of CASF-2016 dataset, which reports the performance of 34 different software/algorithm for the same poses. Figure 1 shows the success rate of predicting the poses with less than 2 Å in the top ranked solutions. The ANI based scoring has been applied when only residues in docking grid are considered as the host in the host–guest interaction, outperforming most of the other 30 scoring functions.

**Fig. 2:**
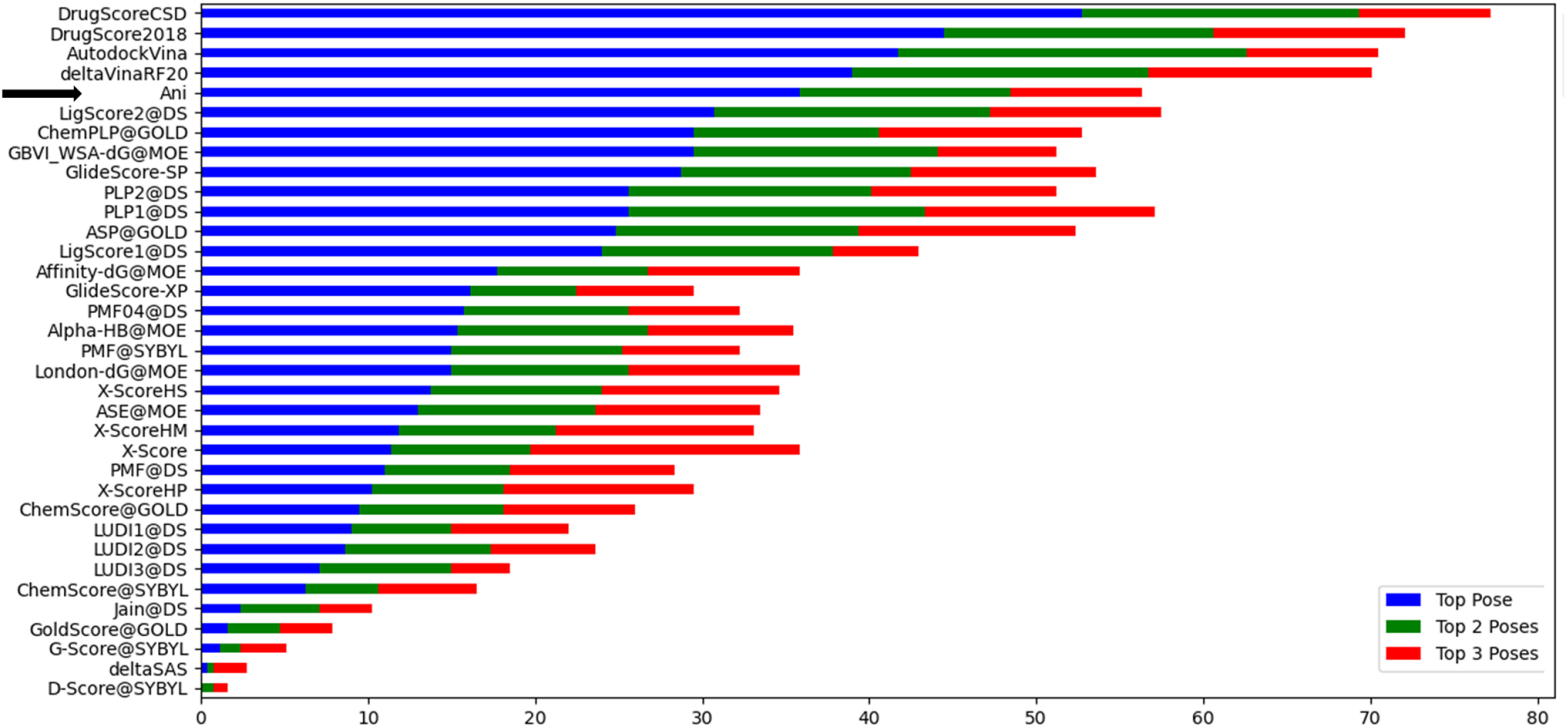
The success of different scoring functions on predicting the first poses as the exact crystal structure. ANI is ranked 5th among 34 functions

In addition, we have also compared the success rate of finding the exact crystal structure in the first ranked solutions (Fig. 2). ANI can still outperform most of the methods finding the true crystal pose among the given decoy poses. It is clear that when a structure is its crystal orientation, ANI can distinguish it much better than most of the methods.

**Fig. 3:**
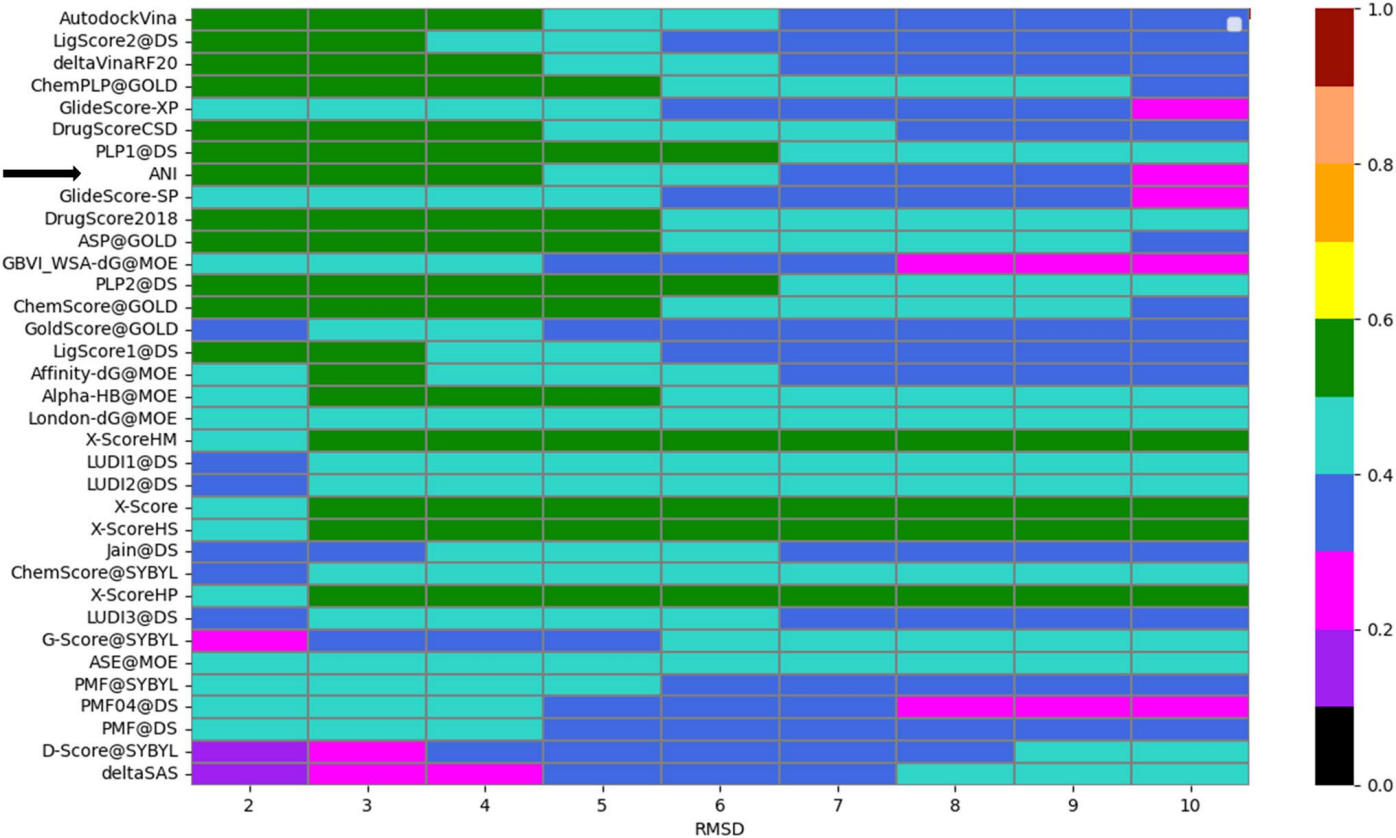
The Spearman correlation coefficients between RMSD and scores for each scoring function being tested. The x-axis indicates the RMSD bins (≤ 2, ≤ 3, etc.) Heat map ranging between 0 and 1 indicates low/high correlations

. In addition to docking power, Yang et al. also defined as “binding funnel analysis” [40], which refers to the docking efficiency for a scoring function. The idea is that the poses that are closer to the crystal structure and those that are far from the crystal structure create a potential energy surface that will look like a funnel. The poses that are closer to the crystal structure will be in the lower energy region of this funnel while the poses that are far from x-ray structure will lie on the higher energy regions. We have performed the same analysis for ANI as well as borrowing the rest of the methods from literature [40] for our set of 254 proteins each with 5 ligands and 100 poses. We have created bins of RMSD windows with 0–2, 0–4,.. 0–10 Å and grouped poses according to these bins. For each RMSD window, the Spearman’s correlation coefficient was calculated Fig. 3. The analysis show that the docking efficiency of ANI based scores are one of the most accurate methods, giving high Spearman’s correlation coefficients in the lower RMSD bins.

### Assessment on SARS-CoV-2 with GOLD

**Fig. 4:**
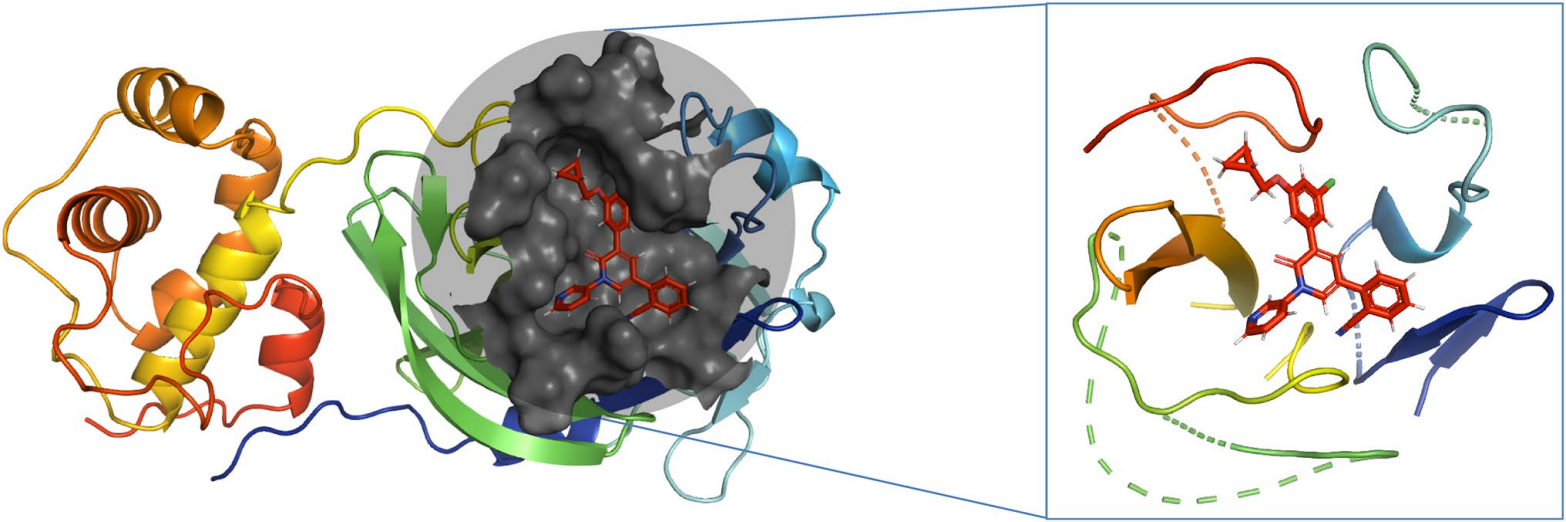
The structure of the SARS-CoV-2 main protease (PDB ID:7L14) used in GOLD docking. The nearby residues around ligand by 10 Å are highlighted.ANI scores were calculated using $\Delta {G}_{ANI}={E}_{complex}-({E}_{protein}+{E}_{ligand})$

In the second part, we assessed the performance of ANI scorings on a specific protein family of SARS-CoV-2 Mpro rather than diverse set of proteins since our focused study is to screen the FDA drugs against this protein. A representative structure of SARS-CoV-2 main protease, used in the docking was shown in Fig. 4.

In order to assess the ANI’s performance using our own docking protocol in GOLD for SARS-CoV-2 main protease-inhibitor complexes, self‐docking is a necessity. Nevertheless, some of the complexes have missing atoms/residues. To overcome this problem, docking all the ligands into a representative receptor structure that is fully resolved (i.e. 7L14) can be a better approach. This would also eliminate possible errors due to using different receptors in comparison of the ligand affinities. However, the complexes available in the PDB show structural differences and residues in the active site show side chain movements. Therefore, selecting only one receptor as a representation for all crystals has also complications to find correlation between docking scores and native binding pose. In order to avoid these error sources, we have selected the most common conformation as a representative structure (i.e. 7L14) for docking all the inhibitors. We have specifically selected 13 other protein-inhibitor complexes available in PDB that have similar scaffold in the binding region. The list of residues in the grid sphere and their RMSD values to the reference structure 7L14 is given in Table S1**.** In all of these crystal structures, the positions of the residues in the active site is protected. This allows to compare the docking of the native ligands of these proteins to a reference protein (7L14).

The top ranked solutions in GOLD and ANI mostly agree and are quite successful in predicting crystal structures. For half of the docked ligands, the top solutions for GOLD and ANI is the same. In addition, the top 3 ranked solutions for GOLD for each of the ligands were also within the first three solutions of ANI.

The success rate of finding the lowest RMSD structure in the top ranked solution was 35.7% for GOLD and 28.6% for ANI. In addition, the average RMSD values of the first poses in GOLD and ANI were 1.48 Å and 1.43 Å, respectively.

**Fig. 5:**
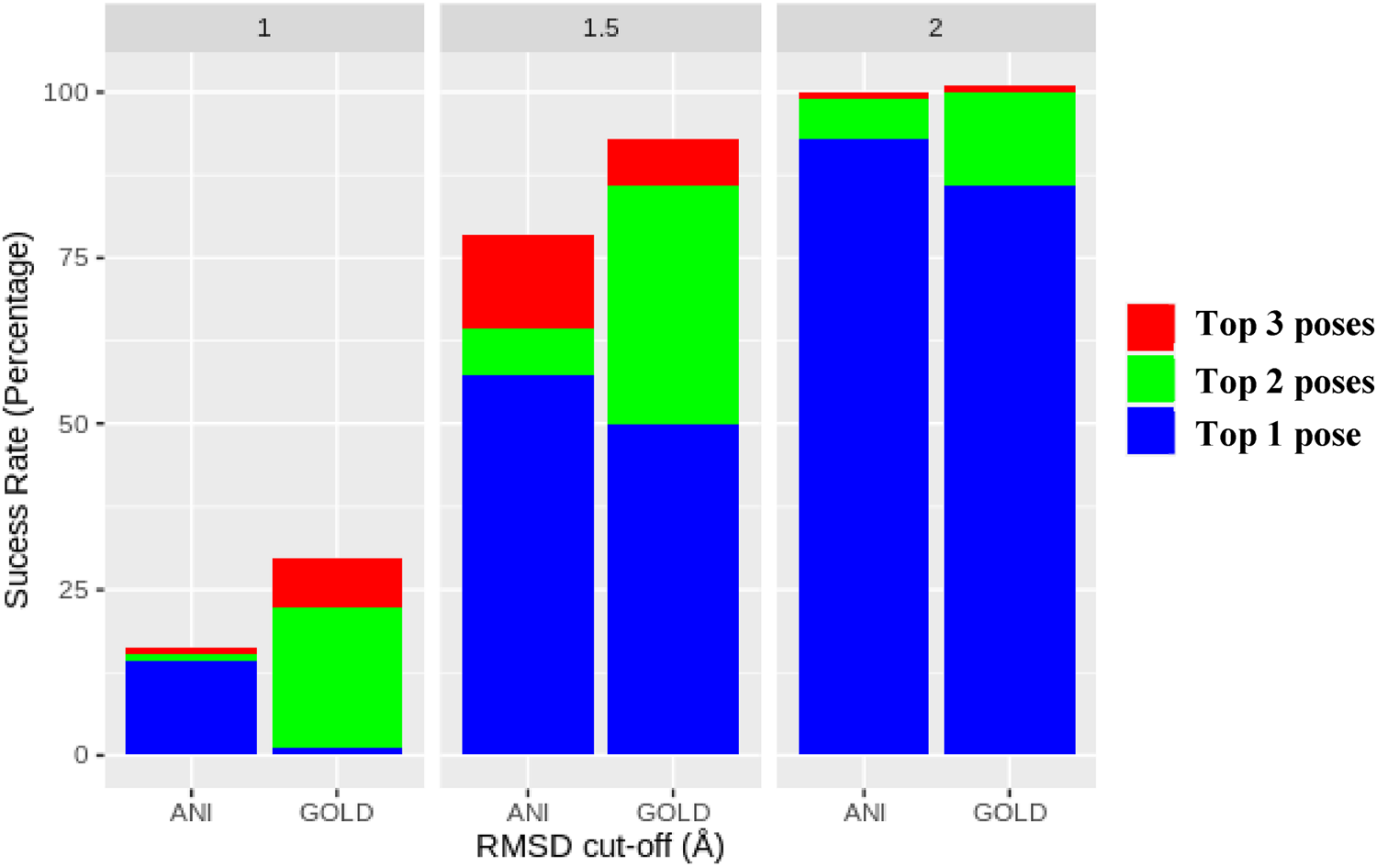
GOLD-PLP and ANI scoring on the poses generated by Gold docking on 14 selected inhibitors of SARS-CoV-2 main protease. ANI shows superiority to GOLD in the top one solution

When we analyzed the top 1 pose, we observed that ANI can find 14.3% of the poses with RMSD ≤ 1.0 Å while GOLD does not find any solution within this cutoff (Fig. 5). For RMSD ≤ 1.5 Å, these values are 57.1% and 50.0% ≤ 1.5 Å, respectively; and 92.9% and 85.7%, respectively for RMSD ≤ 2.0 Å. ANI’s success in the top 1 pose is almost 7% better than GOLD’s success. In general, the top 1 score is considered in the high throughput virtual screening (HTVS) studies aiming to find the putative binders for more advanced computational analysis such as molecular dynamics (MD). Given the fact that the ANI scores are very effective in finding the correct binding conformation at the first pose, it demonstrates the capacity of the ANI based rescoring in virtual screening studies.

**Fig. 6:**
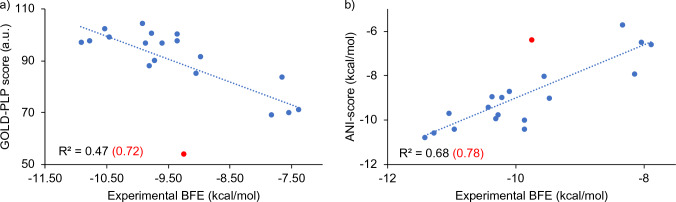
Docking scores generated by **a** GOLD-PLP and **b** ANI with respect to experimental BFEs. ANI scores were produced by scaling with 0.127 as outlined in [41]

The trend between the docking scores of the best poses obtained with the GOLD software and the experimental binding energies shows that the Pearson correlation value is R^2^ = 0.47 (Fig. 6). GOLD scores are arbitrary and the highest scoring values correspond to the highest binding energy (negatively, the lowest binding energy), so the correlation should be inverse. It appears that the GOLD scores are actually in good agreement with the experimental values for the 19 compounds studied. In fact, when the D3F ligand is considered as an outlier, the success of GOLD scores reaches R^2^ = 0.72. This ligand contains multiple nitro (-NO_2_) groups and each N = O bond is assumed double bonds (causing N atom to make 5 bonds) in GOLD due to the difficulty to define the partial atomic charges for these groups as discussed in the GOLD manual [46]. This might explain too low docking scores for this ligand. The ANI interaction energy between the amino acids in the binding site and the ligand showed a better trend with the experimental binding energies. Pearson correlation coefficient was found to be R^2^ = 0.68, outperforming the correlation of GOLD scores. Similarly, when the D3F ligand (red dots in the plots) is considered as an outlier, this coefficient increases up to 0.78.

Instead of assessing the performance of ANI and GOLD scores individually, we have also compared when both methods are combined in consensus scoring. A much higher trend with R^2^ = 0.84 was observed to the experimental values when the top three poses commonly recommended for both GOLD and ANI are considered, clearly supporting the idea of combining these two different methods (GOLD + ANI) as consensus scoring.

### Screening FDA drugs for SARS-CoV-2 Mpro

**Fig. 7:**
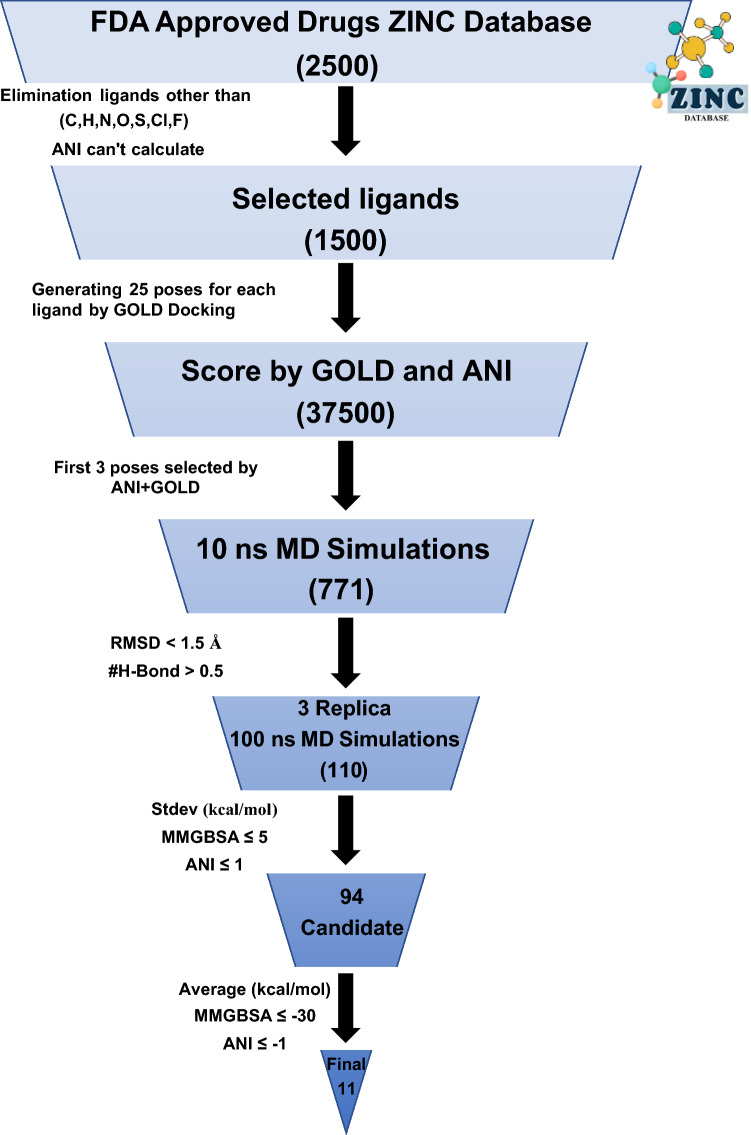
Our unique screening protocol involving consensus scoring of ANI and GOLD, classical MD simulations and consensus BFEs by end-state methods

Our unique screening protocol is given in Fig. 7. MD simulations were initiated using the top three poses obtained from docking. The docking of all FDA-approved drug (2500) molecules in the Zinc database was performed using GOLD. 25 poses for each drug molecule produced by GOLD and rescored with ANI for an additional filtering tool. Since ANI can only perform calculations for C, H, O, N, S, F, and Cl atoms, the number of drugs that are screened decreased to 1460. Our consensus scoring based on GOLD and ANI is as follows: if the top scored three poses generated by GOLD are also ranked in the top three scores with ANI, then these poses are used to MD simulation in the later stage. Otherwise, this complex is discarded. As a result of this filtering process, only 771 out of the best 4380 GOLD docking poses for 1460 drug molecules were also among the top 3 poses according to ANI. All 771 poses belonging to 669 different drug molecules were subjected to MD simulations for 10 ns in the next stage (a total of 7.71 µs of simulations).

**Fig. 8:**
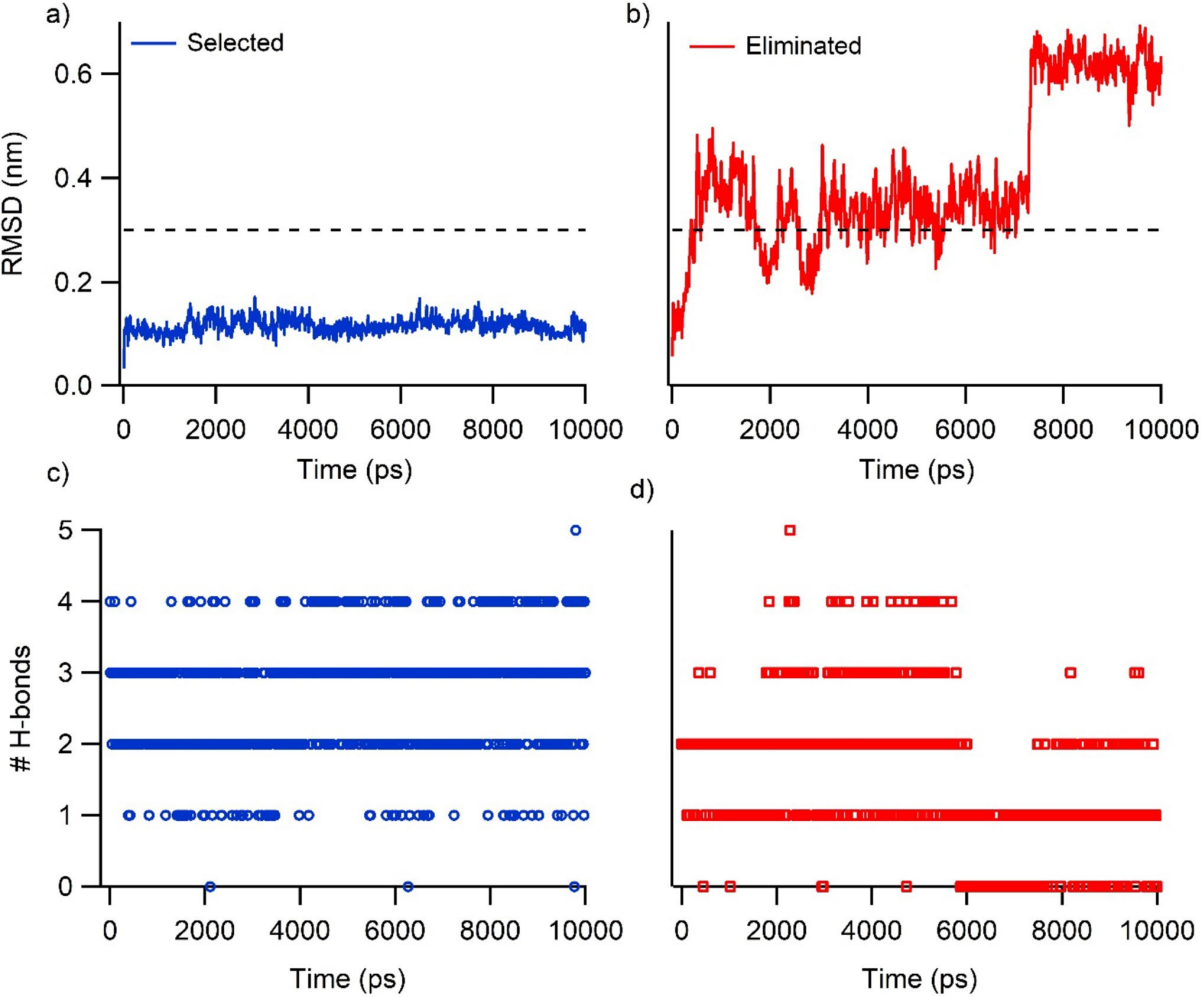
Application of elimination (red)/selection (blue) criteria to MD trajectories. Δ(RMSD) ≤ 3.0 Å for the ligand, and Δ(#H-bonds) ≥ -0.5 between ligand and protein

After the MD simulations, the resulting conformations were evaluated from several aspects to further filter possible SAR-CoV-2 main protease inhibitor candidates. Firstly, it was monitored how far the ligand moved away from the binding site during the MD simulation. If the ligand stably binds to the binding site, it will not move far away from the initial structure. We measured this by fitting the MD trajectory on proteins and calculating the RMSD values of the ligands. The cut-off for the RMSD for this stability test was 3.0 Å. Based on this evaluation, 467 drug molecules remained stable in the binding site. The second criterion for filtering out the ligands is protection of the number of H-bonds. By monitoring the number of H-bonds between the ligands and protein, we eliminated those structures that lose more than 0.5 H-bonds throughout the simulation from our hit list. Figure 8 shows the examples for these criteria. Thus, a total of 346 molecules were identified as potential candidates for binding to the protein. As a final criterion in the classical MD simulations, the stability of the protein–ligand complex was assessed by means of RMSD of back bone atoms of the protein with a cutoff of 0.15 Å (Figure S1). This eliminated further drug poses, leaving over 110 complexes.

In the next step, we performed end-state free energy calculations on remaining 110 compounds each followed by three replica 10 ns long MD simulations (a total of 2.2 µs additional MD simulations). For these selected 110 compounds, free energies were calculated using MMGBSA and ANI_LIE methods (Table S2). Ideally, if a drug-pose interacts with M^pro^ strongly, the free energy values would be much lower. However, if any of the BFE values of the replica simulations is not in the same order as with the other two simulations, then the value predicted is not reliable and should not be trusted. Therefore, we have applied another criterion of standard deviation among the free energies calculated by the replica simulations (5 kcal/mol MMGBSA and 1 kcal/mol ANI_LIE).

In the final step, ANI_LIE and MM-GBSA were used in combination in a way that drug molecules with the lowest binding energies relative to other drugs were suggested by both methods were selected. When the average BFE values of three replica MD simulations for a drug molecule were below −8 kcal/mol by ANI_LIE and −30 kcal/mol by MM-GBSA, the drug molecule was assumed plausible inhibitor for the SARS-CoV-2 M^pro^. The rest of the candidates were eliminated with the assumption that they would not be sufficient for inhibition. Thus, a total of 11 drug molecules remained for which significant binding energy was suggested by 2 different free energy methods. These drug molecules are listed in Table 1.

**Table 1:** The final drug list we recommended in the FDA drug repurposing study to target SARS-CoV-2 Mpro

Name	GOLD score (a.u.)	ANI score (kcal/mol)	MM-PBSA (kcal/mol)		ANI_LIE (kcal/mol)	
			Avg	SD	Avg	SD
Cabazitaxel	58.3	− 9.0	− 44.3	2.6	− 10.8	0.5
Rivaroxaban^a^	61.0	− 8.4	− 40.5	4.8	− 10.4	0.7
Dapagliflozin	69.6	− 10.9	− 33.5	2.3	− 10.3	0.8
Acalabrutinib*	68.3	− 7.9	− 39.3	4.2	− 9.1	0.8
Zaleplon	61.7	− 8.0	− 34.5	2.2	− 8.8	0.3
Sotorasib	62.0	− 8.3	− 31.5	1.5	− 8.7	0.2
Lopac-T-9025	62.9	− 8.5	− 38.3	2.7	− 8.6	0.3
Prucalopride	54.0	− 8.0	− 31.7	0.9	− 8.6	0.1
Lurbinectedin	57.3	− 7.7	− 30.0	1.8	− 8.3	0.6
Thiethylperazine	65.4	− 9.0	− 34.4	0.3	− 8.2	0.0
N-Desmethyl ulipristal acetate	56.6	− 7.0	− 37.3	1.6	− 8.0	0.2

^a^Compounds that have been in clinical trials or suggested in literature

In all three methods, structures with the lowest binding energies were selected as potential drug molecules by consensus. Among the top 5 drug molecules suggested by the common three methods with the highest binding energies, 3 had already been proposed as potential drug molecules in previous theoretical and experimental studies. This confirms the effectiveness of the screening method used here.

Drug molecules listed here are the most plausible SARS-CoV-2 Mpro inhibitors and it is worth performing further experimental analysis to reveal their potency. In our previous studies, we have shown that ANI_LIE values are much more accurate in predicting absolute binding free energies [41]. Therefore, Cabazitaxel, Rivaroxaban and Dapagliflozin might have sub-nanomolar inhibition concentrations for Mpro due to BFEs below − 10 kcal mol^−1^.

## Conclusions

In this study, we have shown the capability of using ANI potentials as a rescoring function in molecular docking. Our benchmarking studies showed that this method can outperform most of the conventional scoring functions. In particular, the prediction of the top 1 solution showed one of the best performances in prediction of the true binding mode. The method can be adopted in any docking software to screen the drug like molecules as potent inhibitors to proteins. As a case study, we introduce our unique screening protocol which incorporates consensus scoring of ANI and GOLD, classical MD simulations and consensus BFEs by end-state methods.

## Supplementary Information

Below is the link to the electronic supplementary material.

Supplementary file1 (XLSX 114 kb)

Supplementary file2 (XLSX 124 kb)

Supplementary file3 (DOCX 280 kb)

## Data Availability

Data is provided within the manuscript or supplementary information files
