# Supplementary material for: Benchmarking ANI potentials as a rescoring function and screening FDA drugs for SARS-CoV-2 Mpro
Source: J Comput Aided Mol Des. 2024 Mar 27;38(1):15. doi: 10.1007/s10822-024-00554-4 (PMC10965596; doi:10.1007/s10822-024-00554-4)
Supplement: Supplementary file 3 — Supplementary file3 (DOCX 280 kb) [file 10822_2024_554_MOESM3_ESM.docx]

Supporting Information for

Benchmarking ANI potentials as a rescoring function and screening FDA drugs for SARS-CoV-2 M^pro^

*Irem N. Zengin^a^, M. Serdar Koca^b,†^, Omer Tayfuroglu^a^, Muslum Yildiz^b^ and Abdulkadir Kocak^a,^[[1]](#footnote-1)^^*

^a^ Department of Chemistry, Gebze Technical University, 41400, Kocaeli/Turkey

^b^ Department of Molecular Biology and Genetics, Gebze Technical University, 41400, Kocaeli/Turkey

**Table S1**. RMSD values of amino acids residing in the docking grid sphere to the reference structure of 7L14

| Res. Num. | 7N44 | 7L10 | 7L11 | 7L12 | 7M8M | 7M8P | 7M8O | 7M8N | 7M8X | 7M8Y | 7M90 | 7M8Z | 7M91 |
| --- | --- | --- | --- | --- | --- | --- | --- | --- | --- | --- | --- | --- | --- |
| 21 | 0.5 | 0.5 | 0.1 | 0.4 | 0.3 | 0.5 | 0.1 | 0.4 | 0.5 | 0.5 | 0.4 | 0.4 | 0.4 |
| 22 | 0.6 | 0.5 | 0.1 | 0.4 | 1.0 | 0.9 | 0.2 | 0.3 | 0.4 | 0.5 | 0.4 | 0.4 | 0.5 |
| 23 | 0.7 | 0.6 | 0.1 | 0.5 | 0.9 | 0.8 | 0.4 | 0.5 | 0.5 | 0.8 | 0.4 | 0.7 | 0.7 |
| 24 | 1.0 | 0.5 | 0.1 | 0.5 | 1.6 | 0.8 | 1.3 | 1.3 | 0.3 | 1.5 | 0.3 | 0.5 | 0.6 |
| 25 | 0.5 | 0.5 | 0.2 | 0.4 | 0.4 | 0.4 | 0.2 | 0.5 | 0.5 | 0.5 | 0.4 | 0.3 | 0.4 |
| 26 | 1.3 | 0.5 | 0.1 | 0.5 | 0.3 | 0.3 | 0.3 | 0.5 | 0.5 | 1.3 | 0.5 | 0.3 | 0.4 |
| 27 | 0.3 | 0.3 | 0.2 | 0.3 | 0.2 | 0.1 | 0.2 | 0.2 | 0.2 | 0.3 | 0.3 | 0.2 | 0.2 |
| 28 | 0.2 | 0.2 | 0.1 | 0.2 | 0.1 | 0.1 | 0.2 | 0.2 | 0.1 | 0.1 | 0.2 | 0.1 | 0.2 |
| 29 | 0.2 | 0.1 | 0.1 | 0.1 | 0.1 | 0.1 | 0.2 | 0.1 | 0.2 | 0.1 | 0.1 | 0.2 | 0.2 |
| 38 | 0.3 | 0.1 | 0.1 | 0.2 | 0.1 | 0.1 | 0.1 | 0.1 | 0.1 | 0.1 | 0.1 | 0.1 | 0.1 |
| 39 | 0.4 | 0.1 | 0.1 | 0.3 | 0.1 | 0.1 | 0.2 | 0.2 | 0.2 | 0.2 | 0.2 | 0.2 | 0.1 |
| 40 | 0.6 | 0.3 | 0.1 | 0.6 | 0.2 | 0.1 | 0.3 | 0.2 | 0.2 | 0.5 | 0.2 | 0.3 | 0.3 |
| 41 | 1.3 | 0.2 | 0.1 | 1.2 | 0.2 | 0.2 | 1.2 | 0.3 | 1.2 | 0.4 | 1.2 | 0.3 | 0.3 |
| 42 | 0.5 | 0.3 | 0.1 | 0.5 | 0.2 | 0.2 | 0.2 | 0.3 | 0.3 | 0.4 | 0.2 | 0.2 | 0.2 |
| 43 | 0.7 | 0.4 | 0.2 | 0.9 | 0.2 | 0.2 | 0.5 | 0.3 | 0.4 | 0.7 | 0.4 | 0.3 | 0.3 |
| 44 | 1.1 | 0.5 | 0.3 | 1.1 | 0.1 | 0.2 | 0.4 | 0.6 | 0.5 | 1.0 | 0.5 | 0.5 | 0.6 |
| 49 | 2.2 | 1.1 | 0.3 | 2.0 | 0.6 | 0.8 | 0.9 | 0.8 | 1.5 | 1.5 | 1.3 | 1.4 | 1.0 |
| 50 | 3.0 | 1.4 | 0.2 | 3.3 | 0.2 | 0.5 | 0.7 | 0.9 | 2.4 | 2.6 | 2.5 | 1.4 | 1.2 |
| 51 | 2.0 | 0.9 | 0.3 | 2.1 | 0.2 | 0.4 | 0.5 | 0.6 | 1.2 | 1.7 | 1.5 | 1.5 | 0.6 |
| 52 | 1.1 | 0.4 | 0.2 | 1.0 | 0.2 | 0.5 | 0.4 | 0.7 | 0.2 | 0.8 | 0.2 | 0.4 | 0.3 |
| 54 | 1.1 | 0.3 | 0.2 | 1.0 | 0.3 | 0.2 | 0.3 | 0.4 | 0.3 | 0.8 | 0.3 | 0.4 | 0.4 |
| 57 | 1.1 | 0.6 | 0.2 | 1.1 | 0.3 | 0.3 | 0.3 | 0.4 | 0.8 | 0.9 | 0.6 | 0.7 | - |
| 85 | 0.6 | 0.2 | 0.1 | 0.5 | 0.2 | 0.1 | 0.2 | 0.1 | 0.2 | 0.4 | 0.3 | 0.2 | 0.3 |
| 114 | 0.1 | 0.2 | 0.0 | 0.1 | 0.1 | 0.2 | 0.2 | 0.2 | 0.2 | 0.2 | 0.1 | 0.2 | 0.2 |
| 116 | 0.2 | 0.3 | 0.1 | 0.2 | 0.1 | 0.1 | 0.2 | 0.2 | 0.3 | 0.1 | 0.2 | 0.3 | 0.3 |
| 117 | 0.1 | 0.2 | 0.1 | 0.1 | 0.1 | 0.2 | 0.2 | 0.1 | 0.3 | 0.2 | 0.2 | 0.2 | 0.2 |
| 118 | 0.3 | 0.3 | 0.1 | 0.2 | 0.1 | 0.2 | 0.2 | 0.2 | 0.3 | 0.2 | 0.2 | 0.2 | 0.2 |
| 119 | 1.2 | 1.2 | 0.1 | 0.2 | 1.1 | 0.2 | 1.2 | 1.1 | 1.2 | 1.1 | 1.1 | 1.1 | 1.1 |
| 120 | 0.2 | 0.3 | 0.2 | 0.2 | 0.1 | 0.2 | 0.5 | 0.3 | 0.2 | 0.1 | 0.3 | 0.2 | 0.2 |
| 126 | 0.3 | 0.3 | 0.0 | 0.4 | 0.2 | 0.3 | 0.2 | 0.1 | 0.5 | 0.4 | 0.1 | 0.3 | 0.3 |
| 135 | 0.2 | 0.1 | 0.1 | 0.2 | 0.2 | 0.3 | 0.4 | 0.2 | 0.2 | 0.3 | 0.2 | 0.1 | 0.1 |
| 136 | 0.3 | 0.1 | 0.1 | 0.2 | 0.2 | 0.2 | 0.3 | 0.2 | 0.2 | 0.3 | 0.2 | 0.1 | 0.1 |
| 138 | 0.3 | 0.1 | 0.1 | 0.3 | 0.4 | 0.1 | 0.1 | 0.2 | 0.2 | 0.3 | 0.2 | 0.2 | 0.2 |
| 139 | 0.2 | 0.2 | 0.1 | 0.2 | 0.2 | 0.1 | 0.1 | 0.2 | 0.2 | 0.3 | 0.2 | 0.2 | 0.2 |
| 140 | 0.2 | 0.1 | 0.1 | 0.2 | 0.3 | 0.2 | 0.2 | 0.2 | 0.2 | 0.2 | 0.2 | 0.1 | 0.1 |
| 141 | 0.3 | 0.3 | 0.1 | 0.3 | 0.3 | 0.3 | 0.6 | 0.4 | 0.4 | 0.3 | 0.2 | 0.2 | 0.2 |
| 142 | 1.7 | - | 1.8 | 2.1 | 1.9 | 1.8 | 1.7 | 1.9 | 0.4 | 1.7 | 1.6 | 1.6 | - |
| 143 | 0.3 | 0.3 | 0.1 | 0.2 | 0.1 | 0.0 | 0.2 | 0.2 | 0.3 | 0.2 | 0.2 | 0.2 | 0.3 |
| 144 | 0.2 | 0.2 | 0.1 | 0.2 | 0.1 | 0.1 | - | 0.2 | 0.2 | 0.1 | 0.2 | 0.2 | 0.2 |
| 145 | 0.3 | 0.2 | 0.1 | 0.2 | 0.1 | 0.2 | 0.2 | 0.2 | 0.1 | 0.2 | 0.2 | 0.2 | 0.2 |
| 146 | 0.2 | 0.1 | 0.1 | 0.1 | 0.1 | 0.1 | 0.2 | 0.2 | 0.1 | 0.1 | 0.2 | 0.1 | 0.2 |
| 147 | 0.1 | 0.1 | 0.1 | 0.1 | 0.1 | 0.1 | 0.2 | 0.2 | 0.1 | 0.1 | 0.1 | 0.1 | 0.2 |
| 161 | 0.2 | 0.1 | 0.0 | 0.1 | 0.1 | 0.2 | 0.1 | 0.2 | 0.1 | 0.1 | 0.2 | 0.1 | 0.1 |
| 162 | 0.3 | 0.1 | 0.2 | 0.2 | 0.1 | 0.2 | 0.2 | 0.3 | 0.1 | 0.2 | 0.2 | 0.1 | 0.1 |
| 163 | 0.2 | 0.1 | 0.1 | 0.1 | 0.2 | 0.2 | 0.2 | 0.3 | 0.1 | 0.2 | 0.1 | 0.1 | - |
| 164 | 0.3 | 0.1 | 0.1 | 0.3 | 0.2 | 0.2 | 0.2 | 0.3 | 0.1 | 0.2 | 0.3 | 0.2 | 0.2 |
| 165 | 0.3 | 0.2 | 0.1 | 0.2 | 0.7 | 0.7 | 1.0 | 0.7 | 0.3 | 1.1 | 0.8 | 0.6 | 0.3 |
| 166 | 0.3 | 0.5 | 0.5 | 0.2 | 0.6 | 0.6 | 0.4 | 0.6 | 0.4 | 0.2 | 0.4 | 0.4 | 0.5 |
| 167 | 0.6 | 0.2 | 0.3 | 0.6 | 0.2 | 0.6 | 0.4 | 0.4 | 0.3 | 0.6 | 0.3 | 0.2 | 0.3 |
| 168 | 1.1 | 0.4 | 0.6 | 1.0 | 0.2 | 0.9 | 0.8 | 0.7 | 0.6 | 1.1 | 0.3 | 0.2 | 0.4 |
| 169 | 1.1 | 0.4 | 0.6 | 1.2 | 0.2 | 1.1 | 0.9 | 0.9 | 0.6 | 1.2 | 0.1 | 0.2 | 0.3 |
| 170 | 0.5 | 0.3 | 0.3 | 0.6 | 0.2 | 0.6 | 0.4 | 0.5 | 0.3 | 0.5 | 0.1 | 0.1 | 0.1 |
| 171 | 0.5 | 0.1 | 0.2 | 0.6 | 0.2 | 0.4 | 0.4 | 0.4 | 0.3 | 0.5 | 0.2 | 0.2 | 0.2 |
| 172 | 0.3 | 0.1 | 0.1 | 0.3 | 0.3 | 0.2 | 0.3 | 0.2 | 0.1 | 0.2 | 0.2 | 0.1 | 0.1 |
| 173 | 0.2 | 0.1 | 0.1 | 0.1 | 0.3 | 0.2 | 0.2 | 0.2 | 0.1 | 0.2 | 0.2 | 0.1 | 0.2 |
| 174 | 0.3 | 0.1 | 0.1 | 0.2 | 0.2 | 0.2 | 0.1 | 0.2 | 0.1 | 0.1 | 0.3 | 0.1 | 0.1 |
| 175 | 0.3 | 0.1 | 0.1 | 0.2 | 0.1 | 0.2 | 0.2 | 0.3 | 0.1 | 0.2 | 0.3 | 0.1 | 0.1 |
| 181 | 0.4 | 0.2 | 0.1 | 0.3 | 0.2 | 0.3 | 0.3 | 0.2 | 0.2 | 0.4 | 0.4 | 0.2 | 0.2 |
| 184 | 0.4 | 0.3 | 0.1 | 0.3 | 0.2 | 0.5 | 0.5 | 0.5 | 0.2 | 0.3 | 0.5 | 0.3 | 0.3 |
| 185 | 0.3 | 0.3 | 0.1 | 0.2 | 0.2 | 0.5 | 0.3 | 0.5 | 0.2 | 0.2 | 0.3 | 0.3 | 0.3 |
| 186 | 0.5 | 0.3 | 0.1 | 0.4 | 0.2 | 0.2 | 0.2 | 0.3 | 0.3 | 0.4 | 0.3 | 0.3 | 0.3 |
| 187 | 0.6 | 0.3 | 0.1 | 0.5 | 0.2 | 0.2 | 0.4 | 0.4 | 0.3 | 0.5 | 0.5 | 0.3 | 0.4 |
| 188 | 2.3 | 1.2 | 0.1 | 2.4 | 0.2 | 0.4 | 0.8 | 0.8 | 1.2 | 2.1 | 1.5 | 0.3 | 0.5 |
| 189 | 1.7 | 2.4 | 1.1 | 1.8 | 2.2 | 1.4 | 1.6 | 1.8 | 1.6 | 1.8 | 1.7 | 1.4 | 2.3 |
| 190 | 1.4 | 1.6 | 0.2 | 1.0 | 1.3 | 0.9 | 1.3 | 1.5 | 1.5 | 0.7 | 1.3 | 1.4 | 1.4 |
| 191 | 1.0 | 1.5 | 0.2 | 0.6 | 0.3 | 1.2 | 0.9 | 1.5 | 0.3 | 0.5 | 0.3 | 0.7 | 0.5 |
| 192 | 1.0 | 0.8 | 0.6 | 1.0 | 0.2 | 1.1 | 1.4 | 1.8 | 0.5 | 0.5 | 0.4 | 0.7 | 0.3 |
| 193 | 2.4 | 0.3 | 0.6 | 2.4 | 0.2 | 1.4 | 2.0 | 2.2 | 1.0 | 1.0 | 0.1 | 0.4 | 0.4 |

**Table S2**. Uzun MD simülasyonları ve serbest enerji hesabı için seçilen 110 ilaç molekülü ve hesaplanan serbest enerji değerleri

|  | GOLD score | MMPBSA | | | ANI | | |
| --- | --- | --- | --- | --- | --- | --- | --- |
| Name |  | 1 | 2 | 3 | 1 | 2 | 3 |
| Cabazitaxel | 58.3 | -44.7 | -41.4 | -46.7 | -11.1 | -10.2 | -10.9 |
| Rivaroxaban | 61.0 | -45.7 | -39.5 | -36.3 | -11.1 | -10.2 | -9.8 |
| Dapagliflozin | 69.6 | -31.3 | -33.2 | -35.9 | -11.2 | -9.6 | -10.2 |
| Miglitol | 43.9 | -21.3 | -22.6 |  | -10.6 | -11.4 | -8.9 |
| Pentostatin | 51.1 | -27.4 | -29.3 | -20.8 | -10.1 | -10.5 | -9.1 |
| Azacitidine | 44.3 | -5.6 | -18.3 | -20.9 | -8.1 | -10.3 | -10.8 |
| Apixaban | 64.2 | -35.1 | -22.6 | -33.6 | -9.8 | -8.1 | -9.7 |
| Tetracycline | 52.7 | -30.2 | -33.4 | -21.5 | -9.4 | -9.6 | -8.5 |
| Acalabrutinib | 68.3 | -44.0 | -38.2 | -35.7 | -10.1 | -8.8 | -8.6 |
| vinorelbine | 50.7 | -46.0 | -49.4 | -32.8 | -9.3 | -9.8 | -8.1 |
| Sulfadoxine | 52.1 | -25.0 | -23.9 | -22.4 | -9.1 | -8.9 | -8.3 |
| Dorzolamide | 43.5 | -14.5 | -24.1 | -15.4 | -8.2 | -9.5 | -8.6 |
| Zaleplon | 61.7 | -36.7 | -32.2 | -34.6 | -8.5 | -8.6 | -9.1 |
| Sarecycline | 58.3 | -26.6 | -28.4 | -30.0 | -8.2 | -8.7 | -9.3 |
| Sotorasib | 62.0 | -31.9 | -32.8 | -29.8 | -8.9 | -8.6 | -8.7 |
| Voriconazole | 51.6 | -25.1 | -32.3 | -20.3 | -9.0 | -8.7 | -8.3 |
| protein_gold_soln_ob257 | 62.9 | -40.4 | -39.3 | -35.3 | -8.6 | -8.9 | -8.3 |
| protein_gold_soln_ob852 | 47.4 | -21.6 | -22.4 | -19.0 | -9.0 | -8.6 | -8.1 |
| protein_gold_soln_ob595 | 53.5 | -24.4 | -23.4 | -21.1 | -8.9 | -8.5 | -8.3 |
| Prucalopride | 54.0 | -32.0 | -32.4 | -30.6 | -8.7 | -8.5 | -8.4 |
| (-)-O-Desmethylvenlafaxine | 53.2 | -23.4 | -23.0 | -24.5 | -9.1 | -8.5 | -8.0 |
| Fluticasone furoate | 53.8 | -34.5 | -28.4 | -20.3 | -9.2 | -7.8 | -8.5 |
| Minocycline | 48.4 | -26.5 | -35.1 | -26.8 | -8.5 | -8.8 | -8.0 |
| Rofecoxib | 50.0 | -26.6 | -26.0 | -26.3 | -8.4 | -8.3 | -8.4 |
| (S)-Praziquantel | 54.0 | -33.2 | -27.8 | -28.7 | -8.8 | -8.0 | -8.3 |
| Meclocycline | 51.7 | -28.7 | -25.1 | -23.7 | -7.9 | -8.7 | -8.4 |
| Desmethyl Tramadol | 49.4 | -21.7 | -19.9 | -26.9 | -8.4 | -8.1 | -8.5 |
| Lurbinectedin | 57.3 | -29.2 | -32.1 | -28.8 | -9.0 | -8.1 | -7.8 |
| (R)-dyphylline | 50.8 | -18.9 | -14.2 |  | -8.9 | -7.6 | -7.8 |
| protein_gold_soln_ob602 | 67.1 | -35.2 | -33.5 | -29.9 | -8.6 | -8.2 | -8.0 |
| Ascorbic Acid | 42.7 | -9.0 | -16.0 | -16.5 | -7.4 | -8.7 | -8.6 |
| mitomycin C | 45.3 | -25.9 | -17.0 |  | -7.9 | -8.1 | -8.6 |
| Thiethylperazine | 65.4 | -34.1 | -34.5 | -34.7 | -8.2 | -8.2 | -8.2 |
| Nelarabine | 49.0 | -23.0 | -20.0 | -6.6 | -9.0 | -8.2 | -7.3 |
| MEBUTAMATE | 36.6 | -12.7 | -20.1 | -17.5 | -7.4 | -8.9 | -8.2 |
| (R)-donepezil | 65.2 | -41.3 | -27.9 | -37.3 | -8.5 | -7.4 | -8.7 |
| Sulfacetamide | 44.7 | -17.7 | -22.8 | -16.1 | -7.9 | -9.0 | -7.7 |
| Norfludiazepam | 47.7 | -22.2 | -23.6 | -21.0 | -7.4 | -8.8 | -8.1 |
| Sulfadiazine | 46.0 | -16.3 | -21.9 | -17.4 | -8.1 | -8.1 | -8.0 |
| N-Desmethyl ulipristal acetate | 56.6 | -38.7 | -35.5 | -37.6 | -8.2 | -7.9 | -8.0 |
| Clopidogrel | 51.9 | -26.5 | -23.9 | -30.5 | -8.1 | -7.6 | -8.3 |
| Podofilox | 53.4 | -28.7 | -29.2 | -31.9 | -8.1 | -8.0 | -7.9 |
| (s)-Trihexyphenidyl | 67.4 | -32.1 | -27.5 | -32.2 | -8.0 | -7.9 | -7.9 |
| Temozolomide | 37.1 | -6.9 | -13.4 | -14.2 | -7.3 | -8.3 | -8.2 |
| Desipramine | 62.7 | -27.8 | -28.5 | -20.2 | -8.3 | -8.3 | -7.3 |
| Benzhydrocodone | 48.3 | -25.5 | -33.8 | -26.2 | -7.9 | -7.9 | -7.8 |
| Larotrectinib | 73.9 | -26.4 | -28.5 | -24.3 | -7.6 | -8.0 | -8.0 |
| (2R,3S)-2-(2,4-difluorophenyl)-3-(4-methylenepiperidin-1-yl)-1-(1H-1,2,4-triazol-1-yl)butan-2-ol | 58.5 | -23.8 | -20.0 | -24.5 | -7.7 | -7.7 | -8.1 |
| Lorlatinib | 53.5 | -29.4 | -27.4 | -21.6 | -7.8 | -7.9 | -7.6 |
| (1S)-2-amino-1-(2,5-dimethoxyphenyl)ethan-1-ol | 46.1 | -15.1 | -20.8 | -17.6 | -7.5 | -8.1 | -7.7 |
| Nandrolone | 45.8 | -25.9 | -29.9 | -20.3 | -7.8 | -8.7 | -6.8 |
| Atovaquone | 56.5 | -29.8 | -27.8 | -31.9 | -7.8 | -7.2 | -8.3 |
| Sulfapyridine | 46.4 | -18.7 | -19.1 | -19.2 | -7.7 | -7.8 | -7.8 |
| Equilin | 46.7 | -26.8 | -25.3 | -25.2 | -7.6 | -7.9 | -7.8 |
| Hydroxyprogesterone caproate | 50.1 | -32.8 | -27.3 | -33.3 | -7.6 | -7.6 | -8.1 |
| SCHEMBL13481056 | 49.2 | -20.1 | -19.5 | -22.6 | -8.1 | -7.1 | -8.0 |
| Sulfamethazine | 47.4 | -21.5 | -21.8 | -21.8 | -7.6 | -7.9 | -7.7 |
| imidazoline | 44.9 | -24.7 | -23.3 | -19.6 | -8.0 | -7.6 | -7.3 |
| O-Desmethyltramadol | 49.5 | -19.3 | -25.8 | -18.4 | -7.0 | -8.9 | -6.9 |
| Hydralazine | 38.0 | -17.3 | -16.8 | -11.5 | -7.9 | -7.8 | -7.2 |
| Menadiol | 45.1 | -16.4 | -15.7 | -16.1 | -7.5 | -7.8 | -7.5 |
| Difluprednate | 54.3 | -27.6 | -27.2 | -22.3 | -7.4 | -8.0 | -7.3 |
| Benzphetamine | 56.7 | -26.6 | -20.4 | -22.0 | -8.0 | -7.3 | -7.4 |
| Estrone - folliculin (has more name) | 46.5 | -27.3 | -28.7 | -25.9 | -7.7 | -7.8 | -7.2 |
| (5S)-5-ethyl-3,5-dimethyl-1,3-oxazolidine-2,4-dione | 30.2 | -17.5 | -17.0 | -17.0 | -7.4 | -7.7 | -7.6 |
| R-epinastine | 44.9 | -17.3 | -23.8 | -18.2 | -7.3 | -7.9 | -7.4 |
| Ondansetron, (3R)- | 62.6 | -27.2 | -20.7 | -25.9 | -7.7 | -7.1 | -7.8 |
| Menadiol | 43.3 | -16.5 | -15.4 | -13.5 | -7.6 | -7.3 | -7.6 |
| Propofol | 41.1 | -18.5 | -21.0 | -19.1 | -7.5 | -7.6 | -7.5 |
| 2-[[(S)-1,2,3,4-Tetrahydronaphthalene]-1-yl]-2-imidazoline | 53.7 | -23.9 | -15.1 | -21.3 | -7.9 | -7.0 | -7.6 |
| Trioxsalen | 43.3 | -23.1 | -21.5 | -22.1 | -7.6 | -7.3 | -7.5 |
| Sulfanilamide | 37.9 | -11.6 | -11.0 | -12.4 | -7.7 | -7.4 | -7.3 |
| (R)-Mepivacaine | 46.8 | -21.9 | -21.4 | -19.8 | -7.4 | -7.5 | -7.5 |
| Sulfanilamide | 34.1 | -13.8 | -12.9 | -13.3 | -7.5 | -7.5 | -7.3 |
| Oxcarbazepine | 44.6 | -16.6 | -17.8 | -18.4 | -7.3 | -7.6 | -7.5 |
| Oxypurinol | 29.9 | -7.2 | -8.3 | -14.3 | -7.2 | -7.3 | -7.7 |
| Lamotrigine | 46.0 | -19.7 | -19.0 | -16.8 | -7.3 | -7.8 | -7.0 |
| Ethylestrenol | 58.3 | -26.3 | -23.4 | -28.1 | -7.0 | -7.4 | -7.6 |
| Sertraline | 55.1 | -17.0 | -22.2 | -25.5 | -7.2 | -7.4 | -7.4 |
| Ethylestrenol | 48.7 | -28.2 | -28.7 | -26.5 | -7.2 | -7.7 | -7.1 |
| Varenicline | 40.0 | -18.7 | -20.5 | -18.1 | -7.0 | -7.6 | -7.5 |
| (R)-Mirtazapine | 45.8 | -22.0 | -22.6 | -23.4 | -7.4 | -7.2 | -7.3 |
| Dapagliflozin | 49.5 | -22.0 | -23.6 | -25.7 | -7.1 | -7.4 | -7.4 |
| Mitotane, (S)- | 46.6 | -24.9 | -26.8 | -27.4 | -7.3 | -7.1 | -7.5 |
| Alprazolam | 49.8 | -21.8 | -21.1 | -26.8 | -7.3 | -7.0 | -7.6 |
| Methoxsalen | 42.1 | -18.5 | -19.7 | -21.8 | -7.0 | -7.3 | -7.5 |
| Cyclizine | 49.5 | -22.0 | -23.0 | -20.3 | -7.3 | -7.3 | -7.2 |
| Norethynodrel | 47.3 | -24.6 | -20.3 | -26.9 | -7.0 | -7.0 | -7.6 |
| Clonidine | 41.8 | -16.8 | -22.7 | -17.0 | -7.3 | -7.3 | -7.0 |
| (R)-Ketamine | 41.3 | -21.9 | -18.2 | -19.2 | -7.6 | -6.9 | -7.1 |
| Bictegravir | 59.9 | -25.7 | -18.6 | -19.8 | -8.0 | -6.7 | -6.8 |
| Anagrelide | 44.8 | -20.6 | -17.1 | -23.6 | -7.1 | -7.2 | -7.3 |
| (S)-Sibutramine | 46.8 | -23.7 | -24.0 | -22.3 | -7.4 | -7.1 | -7.0 |
| Ticlopidine | 56.2 | -20.6 | -21.9 | -25.8 | -7.0 | -7.3 | -7.2 |
| Nicotinamide | 32.4 | -10.9 | -11.1 | -10.4 | -7.2 | -7.2 | -7.0 |
| Nestorone | 45.6 | -21.1 | -22.8 | -23.0 | -7.0 | -7.1 | -7.2 |
| methyl (S)-phenyl[(R)-piperidin-2-yl]acetate | 51.4 | -16.2 | -19.6 | -15.7 | -7.1 | -7.3 | -6.8 |
| (1S)-1-(adamantan-1-yl)ethan-1-amine | 36.6 | -13.4 | -17.4 | -16.7 | -6.9 | -7.1 | -7.1 |
| Isoniazid | 32.8 | -11.1 | -1.4 | -14.2 | -7.2 | -5.8 | -8.1 |
| Pyrimethamine | 45.1 | -23.6 | -21.9 | -22.8 | -6.9 | -6.9 | -7.0 |
| protein_gold_soln_ob1484 | 28.7 | -10.8 | -6.9 | -4.5 | -7.3 | -6.8 | -6.6 |
| Pirfenidone | 47.8 | -10.0 | -14.8 | -15.2 | -6.5 | -6.9 | -6.9 |
| Fomepizole | 27.6 | -10.9 | -3.5 | -7.0 | -7.5 | -6.0 | -6.8 |
| Methyltestosterone | 48.0 | -15.5 | -25.2 | -9.7 | -6.6 | -7.0 | -6.4 |
| trans-Phenmetrazine | 39.1 | -13.1 | -8.7 | -6.6 | -7.1 | -6.6 | -6.3 |
| 4-Methoxyphenol | 35.4 | -10.3 | -13.8 | -1.5 | -7.0 | -7.0 | -5.8 |
| Menadione | 41.3 | -16.5 | -16.7 | -13.0 | -6.5 | -6.8 | -6.4 |
| Deferiprone | 35.6 | -13.8 | -14.7 | -10.2 | -6.5 | -6.6 | -6.6 |
| protein_gold_soln_ob866 | 48.8 | -21.4 | -21.2 | -8.7 | -6.6 | -6.6 | -6.2 |
| Pyrazinamide | 32.0 | -7.5 | -3.5 | -3.8 | -6.8 | -6.3 | -6.2 |


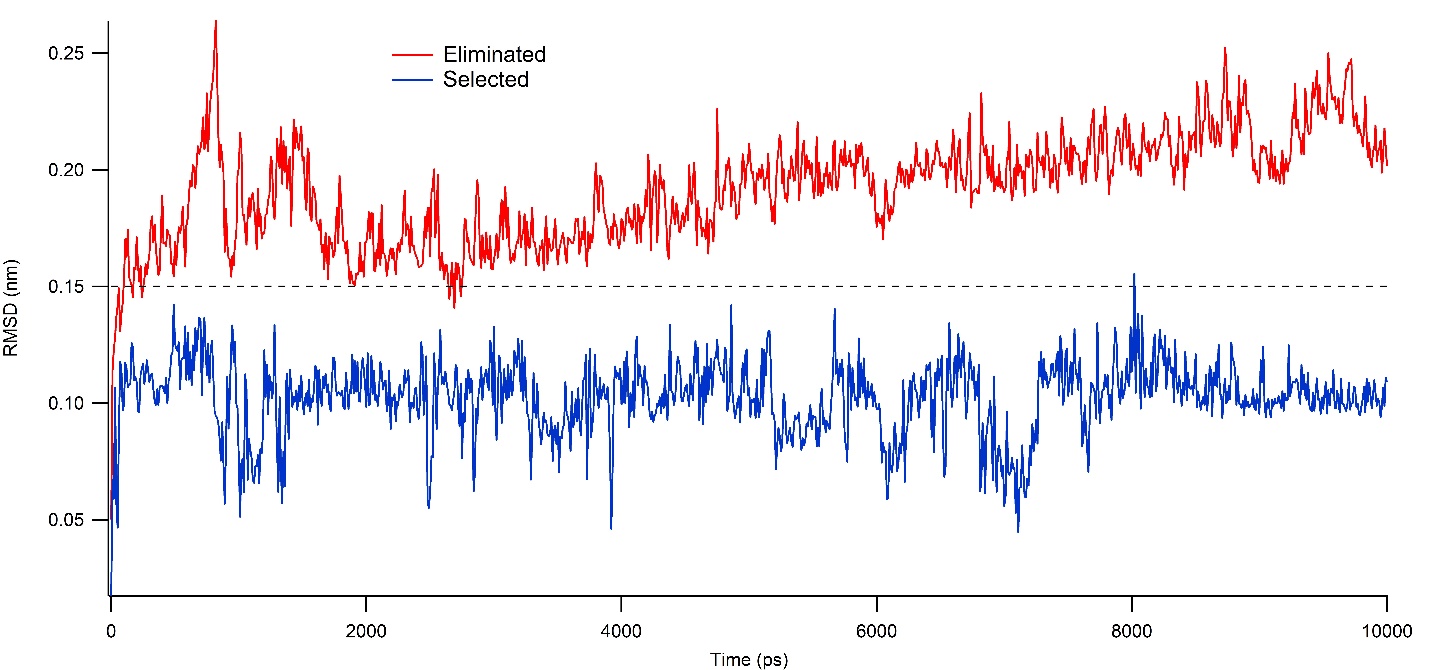


**Figure S1** An example RMSD of protein backbone atoms for selected (blue) and eliminated (red).

1. Corresponding author: Abdulkadir Kocak, kocak@gtu.edu.tr, +902626053083

   ^†^ Current Address: Pfizer - Universidad de Granada - Junta de Andalucía Centre for Genomics and Oncological Research (GENYO), 18016, Granada/Spain [↑](#footnote-ref-1)
